# Enzymatic defense of *Cyperus brevifolius* in hydrocarbons stress environment and changes in soil properties

**DOI:** 10.1038/s41598-020-80854-5

**Published:** 2021-01-12

**Authors:** Paramita Chakravarty, Hemen Deka

**Affiliations:** Environmental Botany and Biotechnology Laboratory, Department of Botany, Gauhati University, Guwahati, Assam 781014 India

**Keywords:** Biotechnology, Environmental biotechnology

## Abstract

Hydrocarbons or crude oil contamination of soil is still a burning problem around the globe. The herbs competent that are to survive in hydrocarbons contaminated habitats have some adaptive advantages to cope up with the adverse situations prevailing in that environment. In the present study, the adaptive response of *Cyperus brevifolius* in the heavily polluted soil with crude oil has been investigated in terms of survivability, changes in productivity, antioxidants, phytochemicals and functional group pro files of the plant species. Besides, changes in enzymes, beneficial bacterial population and physico-chemical conditions of contaminated soil were also studied during 60 days of experimental trials. The results showed significant enhancement in activities of soil dehydrogenase, urease, alkaline phosphatase, catalase, and amylase whereas reduction in cellulase, polyphenol oxidase and peroxidase activities. There was a significant increase in nitrogen fixing, phosphate and potassium solubilizing bacterial population, improvement in physico-chemical conditions and a decrease in total oil and grease (TOG) levels. Besides there was significant variations in the productivity parameters and antioxidant profiles of *Cyperus brevifolius* in hydrocarbons stress condition suggesting enzymatic defense of the herb. The fourier-transform infrared (FT-IR) analysis indicated uptake and metabolism of some hydrocarbon components by the experimental plant from the hydrocarbons polluted soil.

## Introduction

Soil pollution due to crude oil contamination is a burning problem around the globe. This problem is likely to increase due to dependence on petroleum and other oil products as a prime source of energy all over the world^1^. The accidental release of oil/hydrocarbons causes pollution and alters the physical, chemical and biological conditions of the soil. The uptake of oil/hydrocarbon pollutants by plants results in food chain contaminations while downward movement of these pollutants causes ground water pollution^2^. As a whole this is very harmful for human health and environment.

The phytoremediation has been proven as an effective technique for remediation of crude oil contaminated soil^2,3^. It has also been reported as a green clean technology and is alternative to physical and chemical methods of remediation^4^. However, success of phytoremediation basically depends on the type of plants used in the process besides in most cases it is even site specific. Moreover, due to better adaptability as supported by their fibrous root systems; herbaceous and annual plants have been reported as suitable candidates for remediation of crude oil contaminated soils. It has been frequently reported that the herbaceous and/or annual plant species show better adaptive response in polluted sites as compared to tree seedlings or saplings^4–6^. These plants have the efficiency in cleaning up the polluted soils at the early stage of their growth^7^. Several works have highlighted the success of annual/perennial herbs for phytoremediation of oil-contaminated soil throughout the globe^7,8^.

The different species of *Cyperus* has been reported as an effective candidate for remediation of crude oil polluted soil due to their quick and proliferate growth in the contaminated environment^5,9–11^. According to a report, extensive root systems support large number of microbial populations in oil contaminated soil systems leading to produce several enzymes for degradation of petroleum contaminants and evapotranspiration of volatile hydrocarbons^11^. The species of *Cyperus* are characterized by their fibrous root systems and do not need long term maintenance in the stress environment which make them a suitable candidate for being used in the phytoremediation process. The efficacy of this herb species has been tested for removal of oil and grease and it can survive in 50–80 g/kg of crude oil spiked soil^5,12,13^. Even, removal of hydrocarbons from an initial range of 65–75 g/kg was also found feasible by *Cyperus brevifolius* in crude oil contaminated field^10^. Nevertheless, the study focusing on the response of *Cyperus brevifolius* in heavily polluted soil with crude oil is still limited and need to be addressed adequately. Moreover, the success of any plant-based technique depends upon the survivability of plants in heavily contaminated soil under field condition. In this context, the understanding of plant’s adaptive response as well as enzymatic defense mechanism during crude oil reclamation process is necessary and accordingly a planned study is required.

The herb *Cyperus brevifolius* has been reported to grow abundantly in the sites that are heavily contaminated with crude oil^5^. Hence, it is hypothised that this herb species has some adaptive advantages to cope up with the adverse situation that are prevailing in the oil contaminated habitats. Besides, it is also assumed that *Cyperus brevifolius* have ability to exhibit strong enzymatic defense to avoid hydrocarbon associated abiotic stress and also improves soil condition by up taking/detoxifying the pollutants from the contaminated soil. Keeping this in mind, the present work was designed to investigate the enzymatic defense process of *Cyperus brevifolius* in aged/heavily polluted soil with crude oil. During the study, the changes in soil enzyme activities and beneficial microbes that include the total population of nitrogen fixing, phosphate and potassium solubilizing bacteria were also investigated to know their relationship with soil as well as plant and to correlate the changes in NPK status in the contaminated soil. Besides, the survivability, productivity, phyto-chemical contents as well as changes in functional groups of the plant species, oil and grease contents and physico-chemical conditions of the contaminated soil have also been investigated during the study.

## Results

### Survivability of Cyperus brevifolius in crude oil polluted soil

Initially ten individuals of *Cyperus brevifolius* was introduced separately in ten respective pots that contains crude oil polluted soils of oil field. The mortality of introduced plant was observed during the entire experimental duration. After 43 days of observation it was found that five numbers of individuals of *Cyperus brevifolius* has survived out of the ten introduced initially till the end of experimental trials. Thus the survivability rate of the *Cyperus brevifolius* in the crude oil contaminated soil was found to be 50%.

### Changes in the soil properties

#### Physico-chemical changes

The physico-chemical profiles in the crude oil-contaminated soil samples were analyzed at the beginning and by the end of the experimental trials. The results have been presented in Table 1. The initial pH value indicates high acidic condition in the contaminated soil which has reduced significantly by 23.93% in T3 after 60 days of treatment*. *There was no significant change in pH level in T2 (Table 1). The electrical conductivity (EC) and water holding capacity (WHC) values have also increased in T3 than the initial level as obtained in T1. The increase in conductivity was found 3.04 fold in T3 as against marginal deviation in the values in T2 (Table 1). Similarly, an increase (31.39%) in WHC was recorded in T3 when compared with the initial level (T1). There were no significant changes in WHC content in T2. The reduction in TOC was recorded as 12.1% in T3 and 5.11% in T2 than the initial level as recorded in T1. The total KJELDAHL nitrogen (TKN) content has decreased but available phosphorus (AP) and total potassium (TK) increased in T3. The decrease of TKN was 1.2 fold whereas the increase was recorded 1.3 fold for AP and twofold for TK. Similar increasing trend (1.3 folds) for AP and TK was observed in T2 although no significant changes have been noticed in TKN level.

**Table 1:** Showing the changes in physico-chemicals and total oil and grease profiles in contaminated soil at the beginning and after 60 days of introduction of *Cyperus brevifolius.*

Parameters	T1	T2	T3
pH	4.27 ± 0.081a	4.289 ± 0.024a	5.292 ± 0.019b
Conductivity (ms/cm)	0.08 ± 0.007a	0.078 ± 0.010a	0.243 ± 0.009b
Water holding capacity (%)	9.86 ± 0.701a	9.89 ± 0.561a	41.25 ± 1.903b
Total organic carbon (%)	18.754 ± 1.196a	13.64 ± 0.521b	6.659 ± 0.891d
Total nitrogen (mg/kg)	890 ± 0.004a	860 ± 0.012a	720 ± 0.008b
Available phosphorus (mg/kg)	30 ± 1.700a	40.2 ± 0.601b	38.60 ± 0.201c
Total potassium (mg/kg)	272.563 ± 8.011a	345.33 ± 2.081 k	537.67 ± 4.132d
TOG (g/kg)*	111.333 ± 5.271a	110.503 ± 3.133a	92.001 ± 6.052c
**Soil texture**			
Sand (%)	83.34 ± 0.321p	82.07 ± 0.820p	77.87 ± 0.192 k
Silt (%)	9.71 ± 0.262 m	8.13 ± 0.075 m	6.147 ± 0.313 s
Clay (%)	6.95 ± 0.281a	8.89 ± 0.530b	13.55 ± 0.219c

Mean value ± SD, n = 3; the different letters within the same row represent the significant differences of the values (ANOVA, LSD test, *P* ≤ *0.05*).T1- Crude oil contaminated soil (Initial); T2- Crude oil contaminated soil without *Cyperus brevifolius*; T3- Crude oil contaminated soil + *Cyperus brevifolius.*

*Total oil and grease contents.

The gravimetric analysis revealed 17.36% reduction in total oil and grease (TOG) contents in T3 as against the initial values obtained in T1. There were no significant changes in TOG level in T2. The results of soil texture are presented in Table 1. In T3, the percentage values of clay content has increased significantly as against the reduction in the values of sand and silt content by the end of the experimental trial. There were marginal changes for sand and silt contents in T2 (Table 1) although the percentage values of clay content increased significantly after 60 days of experimental trials.

#### Changes in soil enzyme activity and beneficial bacterial population

**Figure 1:**
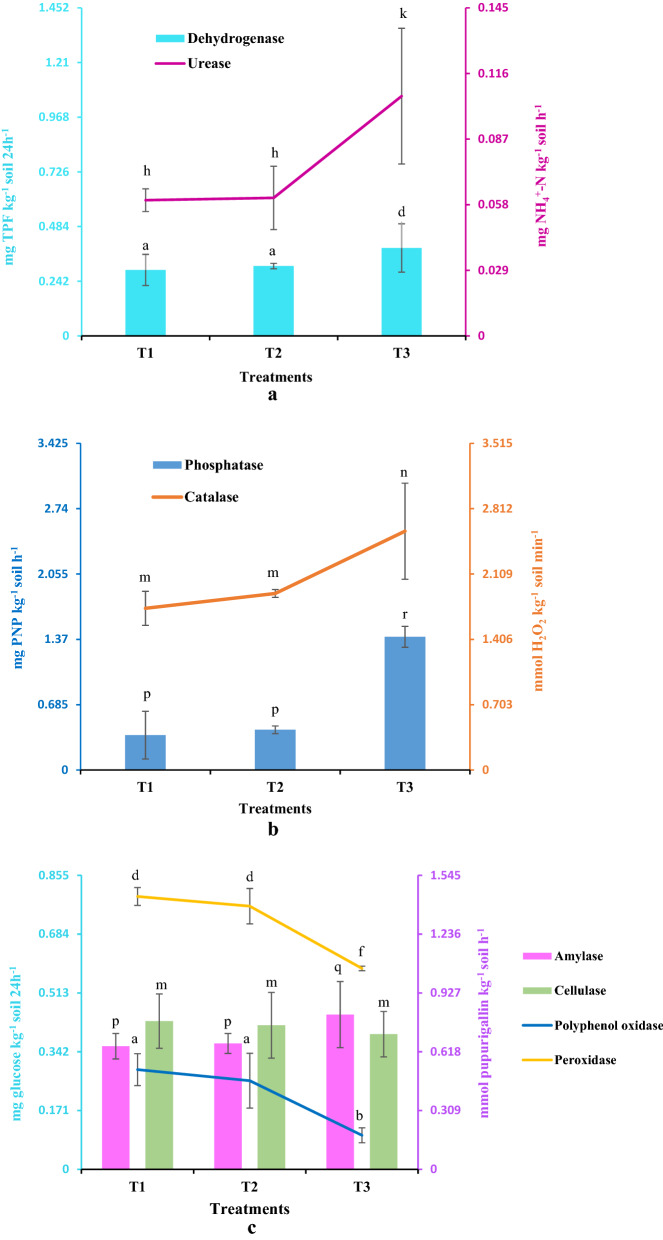
(**a**) Showing the changes in dehydrogenase and urease activities in crude oil contaminated soil at the beginning and by the end of the experimental trials. Values are mean, n = 3, error bars indicate SD. Different letters above error bars stand for significant differences of the values for a particular enzyme among the different treatments (ANOVA, LSD test, *P* ≤ *0.05*). Black axis-for treatments details, Light blue axis-for dehydrogenase activity, Purple axis-for urease activity. T1- Crude oil contaminated soil (Initial), T2- Crude oil contaminated soil without *Cyperus brevifolius*, T3- Crude oil contaminated soil + *Cyperus brevifolius*. (**b**) Showing the changes in alkaline phosphatase and catalase activities in crude oil contaminated soil at the beginning and by the end of the experimental trials. Values are mean, n = 3, error bars indicate SD. Different letters above error bars stand for significant differences of the values for a particular enzyme among the different treatments (ANOVA, LSD test, *P* ≤ *0.05*). Black axis-for treatments details, Blue axis-for phosphatase activity, Brown axis-for catalase activity. T1- Crude oil contaminated soil (Initial), T2- Crude oil contaminated soil without *Cyperus brevifolius*, T3- Crude oil contaminated soil + *Cyperus brevifolius*. (**c**) Showing the changes in amylase, cellulase, polyphenol oxidase and peroxidase activities in crude oil contaminated soil at the beginning and by the end of the experimental trials. Values are mean, n = 3, error bars indicate SD. Different letters above error bars stand for significant differences of the values for a particular enzyme among the different treatments (ANOVA, LSD test, *P* ≤ *0.05*). Black axis-for treatments details, Light blue axis-for amylase and cellulase activities, Violet axis-for polyphenol oxidase and peroxidase activities. T1- Crude oil contaminated soil (Initial), T2- Crude oil contaminated soil without *Cyperus brevifolius*, T3- Crude oil contaminated soil + *Cyperus brevifolius*.

The results of soil enzyme activities are presented graphically in Fig. 1a,b,c. The results showed that activities of dehydrogenase, urease, alkaline phosphatase, catalase and amylase in T3 soil samples have increased after 60 days of experimental trials. However, at the same time, there was a decrease in peroxidase and polyphenol oxidase activities in the T3 soil samples by the end of the experimental trials. No significant changes have been noticed for all the studied enzymes in T2 samples. The values of dehydrogenase activity in T1 sample was 0.292 ± 0.069 mg TPF kg^−1^ soil 24 h^−1^ which has increased significantly in T3 sample and recorded as 0.389 ± 0.107 mg TPF kg^−1^ soil 24 h^−1^ (Fig. 1a). The urease activity in T1 samples was recorded 0.06 ± 0.005 mg NH_4_^+^-N kg^−1^ soil h^−1^ which has increased up to 0.106 ± 0.03 mg NH_4_^+^-N kg^−1^ soil h^−1^ in T3 sample (Fig. 1a). The initial value of alkaline phosphatase activity as found in T1 sample was 0.366 ± 0.25 mg PNP kg^−1^ soil h^−1^ whereas in T3 sample the value was 1.397 ± 0.11 mg PNP kg^−1^ soil h^−1^ by the end of 60 days (Fig. 1b). The catalase activity in T3 sample has also increased significantly and recorded as 2.57 ± 0.517 mmol H_2_O_2_ kg^−1^ soil min^−1^ as against the initial level found in T1 sample (Fig. 1b). Similarly, amylase activity had also increased significantly in T3 sample and found to be 0.450 ± 0.096 mg glucose kg^−1^ soil 24 h^−1^ as against the initial value (0.358 ± 0.037 mg glucose kg^−1^ soil 24 h^−1^) of T1 sample (Fig. 1c). There was no significant deviation in cellulase activity values irrespective of the treatments trials (Fig. 1c) while both polyphenol oxidase and peroxidase activities were decreased significantly in T3 samples (Fig. 1c).

The total number of beneficial microbial population that includes nitrogen-fixing, phosphate and potassium solubilizing bacteria has been expressed in terms of colony-forming unit per gram soil (CFU g^−1^soil) and are presented in Table 2. The results showed that there was 3.29 fold increases in N_2_ fixing population in T3 sample whereas it was 1.3 fold in T2 after 60 days of experimental period. Similarly, the increase in P solubilizing bacterial population in T3 was 8.73 fold as against 1.34 fold in T2 sample. Further, for K solubilizing bacteria the increase was 6.17 fold in T3 and 1.01 fold in T2 samples.

**Table 2:** Showing the changes in beneficial bacterial population in crude oil contaminated soil after 60 days of experimental trials employing *Cyperus brevifolius.*

Treatments	Nitrogen-fixing	Phosphate solubilizing (× 10^7^ CFU/g soil)	Potassium solubilizing
T1	14.67 ± 0.23a	2.67 ± 0.53a	3.08 ± 0.45v
T2	19.11 ± 0.14a	3.59 ± 0.09a	3.12 ± 0.06v
T3	48.33 ± 0.09b	23.30 ± 0.22d	19.0 ± 0.87y

Mean value ± SD, n = 3; the different letters within the same column represent the significant differences of the values (ANOVA, LSD test, *P* < *0.05*). T1- Crude oil contaminated soil (Initial); T2- Crude oil contaminated soil without *Cyperus brevifolius*; T3- Crude oil contaminated soil + *Cyperus brevifolius.*

### Changes in plant characteristics

#### Changes in plant productivity parameters

The results of plant productivity parameters that include leaf area index (LAI), chlorophyll and dry biomass contents are presented in Table 3. The LAI of *Cyperus brevifolius* was increased by28.47% in T3 and 61.64% in T4 samples. The results of chlorophylls showed that chlorophyll a, b and total chlorophyll values decreased significantly by 37.83%, 59.16% and 31.37% respectively in the harvested plant samples of T3 after 60 days. On the other hand, in case of plant samples of T4, there was an increase in chlorophyll a, b and total chlorophyll values by 18.38%, 23.32% and 12.02% respectively by end of experimental trials. The results also showed a significant increase in dry biomass of both shoot and root by the end of the experimental trials (Table 3). The increase in dry biomass of shoot was found maximum (168.97%) in case of T4 whereas dry biomass value of root was maximum (204.90%) in T3 sample.

**Table 3:** Showing the changes in productivity parameters and phytochemical contents of *Cyperus brevifolius.*

Parameters	Treatments		
	Ti	T4	T3
**Productivity**			
i) Leaf Area Index	1.001 ± 0.009a	1.618 ± 0.015b	1.286 ± 0.0122c
ii) Dry Biomass (g)			
Shoot	0.029 ± 0.006p	0.078 ± 0.025q	0.055 ± 0.036q
Root	0.102 ± 0.09 m	0.154 ± 0.076n	0.311 ± 0.113 k
iii) Chlorophyll (µgml^−1^)			
*Chl-a	5.567 ± 0.100 h	6.590 ± 0.016 k	3.461 ± 0.011p
*Chl-b	0.862 ± 0.081b	1.063 ± 0.067c	0.352 ± 0.002d
Total chlorophyll	12.987 ± 2.056a	14.548 ± 0.046b	8.913 ± 0.019c
**Phytochemical contents**			
i) Phenol (mg gallic acid/g of extract)	–	0.691 ± 0.009a	0.828 ± 0.041b
ii) Flavonoid (mg quercitin/g of extract)	–	12.256 ± 0.083p	12.754 ± 0.026q

Mean value ± SD, n = 3; the different letters within the same row represent the significant differences of the values (ANOVA, LSD test, Paired t-test, *P* < *0.05*). Ti- seedlings of *Cyperus brevifolius* grown in soil bed initially for the experiment; T4- Non-contaminated soil + *Cyperus brevifolius*; T3- Crude oil contaminated soil + *Cyperus brevifolius.*

*chl-a- Chlorophyll-a, *chl-b- chlorophyll-b.

#### *Changes in plant’s *in vitro* antioxidant activity and phytochemical contents*

**Figure 2:**
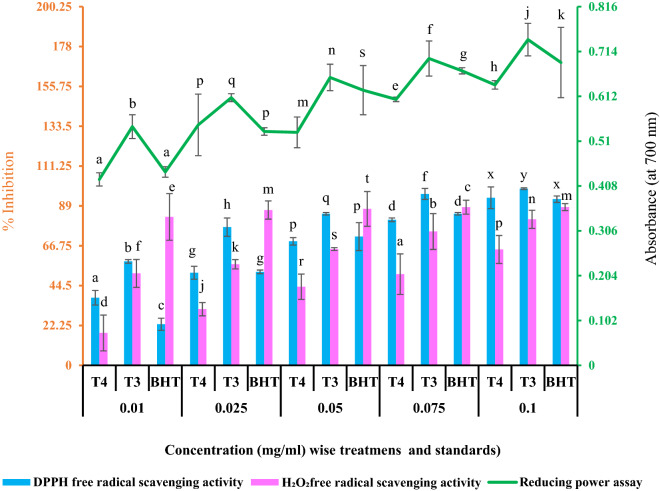
Showing the changes in DPPH, H_2_O_2_ free radical scavenging activities and reducing power assay of *C. brevifolius* extracts grown in crude oil contaminated soil and control soil. Values are mean, n = 3, error bars stand for SD. Significant differences of the values for a particular parameter at a particular concentration for different treatments and standard are indicated by different letters (ANOVA, LSD test, *P* ≤ *0.05*). Black axis-for concentration wise treatments and standards, Brown axis-for DPPH and H_2_O_2_ free radical scavenging activity, Green axis-for reducing power assay.T4- Non-contaminated soil + *Cyperus brevifolius,* T3- Crude oil contaminated soil + *Cyperus brevifolius*, BHT- Butylated hydroxytoluene.

The plant’s in vitro antioxidant status such as reducing power assay, DPPH and H_2_O_2_ free radical scavenging activity have been shown graphically in Fig. 2. The results were also compared with standards i.e. Butylated hydroxytoluene (BHT). Besides, the polyphenol and flavonoid profiles of the plant extracts are presented in Table 3. The results showed that the reducing power of the plant extract was increasing with the increasing concentration. The order of the result was found as- T4 < BHT < T3 (Fig. 2) suggesting enzymatic adaptive response of the plant in a stress environment. Further, the plant extract also showed a concentration-dependent scavenging activity against DPPH and H_2_O_2_ radicals. The IC50 value of the plant extract for DPPH scavenging in T3 sample was found to be lower (0.018 ± 0.001 mg/ml) when compared with the T4 (0.072 ± 0.004 mg/ml) samples. The results when compared with the standard solutions of BHT showed IC50 value of 0.037 ± 0.001 mg/ml. Similar trend was also noticed in case of H_2_O_2_ free radical scavenging activity. The lower IC50 value (0.029 ± 0.003 mg/ml) was found for the T3 as against the T4 (0.056 ± 0.002 mg/ml) and standard BHT (0.032 ± 0.001 mg/ml). The values of phenol and flavonoid content were increased significantly for the experimental plant extract in T3 as against the T4 samples (Table 3).

#### FT-IR analysis

FT-IR spectroscopy was conducted to identify the changes in functional groups of the experimental plant in crude oil associated stress conditions. A comparison has been made with the plant sample from non-contaminated soil i.e. T4. The results of FT-IR have been presented in Table 4. The results showed several common peaks and bands at respective wave number (cm^−1^) representing various functional groups that are present in both the plant samples from T3 and T4 treatments. However, there were some distinct variations in sharpness and intensities of the peaks and bands between the plant samples of T3 and T4 treatments. The results confirmed the presence of phenols and flavonoids (3650–3150 cm^−1^), methylene (2926, 2853 cm^−1^), aldehydes (1737 cm^−1^), alkenes-cyclohexenes (1635 cm^−1^), aromatics (1432 cm^−1^), alkyl aryl ethers-anisole (1250 and 1040 cm^−1^), alcohols, esters, ethers, carboxylic acids (1031 cm^−1^), aliphatic chlorides (780, 696 cm^−1^) and iodide (538 cm^−1^) compounds (Table 4). The broad band between 3650 and 3150 cm^−1^ due to O–H stretching indicates the presence of flavonoids/phenolic compounds was found in both T3 and T4 plant samples. However, less broadness and more intensity of this band is evident in the plant sample of T3 (Table 4)*.* The peaks related to C–H stretching of methylene appear at 2926 cm^−1^ and 2853 cm^−1^ with marginally increased intensities in T3 than T4. Another band at 1737 cm^−1^ associated with C=O stretching of aldehydes were more clear and intense in T3 than in T4. Again C=C stretching of alkenes such as cyclohexene at 1635 cm^−1^ were noticed in both samples, but the peak intensity was marginally higher in T3 samples as against T4. The spectrum of 1432 cm^−1^ which may be due to C=C stretching of aromatics was apparent and distinct in the sample from T3. Another two peaks at 1250 and 1040 cm^−1^ indicating C–O stretching of alkyl aryl ethers more likely anisole were found more sharply and dominantly in T3 when compared with T4. Whereas the peaks at 1031 cm^−1^, 780 cm^−1^, 696 cm^−1^ and 538 cm^−1^ though present in both the samples, it was more distinct and intense in T4 sample than T3 indicating C–O stretching of alcohols/ethers/esters/carboxylic acids, C–Cl stretching of aliphatic chloride and C–I stretching of iodide respectively. From the above results, it was observed that there was both increase as well as decrease in intensities of various peaks/bands between the studied plant samples for T3 and T4.

**Table 4:** Showing absorbance of IR spectra along with their assignments in the tested plant samples grown on T3 and T4 treatments.

Wave number (cm^−1^)	Assignments	Samples
3650–3150	O–H stretching broad band of flavonoids/phenolic compounds	Present in both the samples, more intense in T3 samples
2923, 2853	C–H stretching representing methylene	Present in both the samples, marginally intense in T3 samples
1737	C=O stretching of aldehydes	Clear and intense in T3 sample than T4
1635	C=C stretching of alkenes such as cyclohexane	Present in both the samples, little intense in T3 samples
1432	C=C Stretching of aromatics	More clear and intense in the sample from T3
1250, 1040	C–O stretching of alkyl aryl ethers such as anisole	Dominantly present in T3 sample
1031	C–O stretching of alcohols/ethers/esters/carboxylic acids	Present in both samples, sharp and intense in sample from T4
780, 696	C–Cl stretching aliphatic chlorides	Clear and intense in T4
538	C–I stretching of iodides	Sharp and more intense in samples of T4

T3- Crude oil contaminated soil + *Cyperus brevifolius*; T4- Non-contaminated soil + *Cyperus brevifolius.*

## Discussion

### Survivability of Cyperus brevifolius

The adverse effects of crude oil/petroleum hydrocarbons on soil physico-chemicals and biological properties have already been reported^1,11^. The crude oil contamination clogged the soil pore spaces, reduces soil porosity, aeration, water infiltration and increases the bulk densities which ultimately reduces water holding capacities and hampers the nutrients availability for plants^14,15^. This in turn also hampers the survival of plants in the oil polluted habitats. The present observation reveals fifty percent death of the individuals of *Cyperus brevifolius* which can be attributed to the water and nutrient deficiency in the soil that are highly polluted with crude oil/hydrocarbons. The findings agree with the previous works of Adieze et al.^16^ who had reported 75%, 43.8%, 56.3% and 50% survivability rate for *Panicum maximum, Zea mays, Centrosema*sp.*, and Pueraria*sp. respectively over 10 weeks after increasing the crude oil concentration up to 10% (w/w). In a study, Han et al*.*^14^ reported that crude oil pollutants can obstruct the uptakes of water and nutrients in plants which causes membrane injuries due to accumulation of reactive oxygen species (ROS).This inhibits the photosynthesis and transpiration process thereby suppressing the growth and development and even sometimes death of the plants. Here, it is hypothesized that survivability as well as mortality of *Cyperus brevifolius* is related to specific defense of the plant species which vary individual to individual depending on the soil conditions. Nevertheless, further planned study is required to prove this hypothesis.

### Changes in the soil properties

#### Physico-chemical characteristics and TOG contents

The present findings regarding soil physico-chemical and biological parameters clearly indicate the adverse effects of crude oil on soil system. The crude oil contamination clogged the soil pore spaces, holds the soil particles together and reduces soil porosity, aeration, water infiltration and increases the bulk densities which ultimately reduce water holding capacity and destroys the soil textures^15^. Besides, lower soil porosity and water holding capacity are also associated with reduction in microbial population, poor mineralization and nutrients availabilities in the soil^14^. Nevertheless, there was improvement in soil physico-chemicals and biological conditions by end of the experimental trials. The shifting of soil pH from high to low acidic condition at the end of the study may be due to either microbial degradation of crude oil pollutants or because of increasing metabolic activities in the soil by plant growth and associated microbial communities^5^. Again present findings for changes in electrical conductivity (EC) are an agreement with the previous works of Tanee and Akonye^17^ who have reported an increased level of EC in the plant’s treated contaminated soil. Electrical conductivity is an important parameter for soil health as it is associated with soil structure and water infiltration^18^. The EC value within 0.11–0.57 ms/cm is suitable for agricultural activities^19^. Similarly, there was enhancement in water holding capacity (WHC) in the contaminated soil after treatment by *Cyperus brevifolius.* The enhanced values of EC and WHC could be attributed to the plant–microbe interactions which ultimately degrade the oily layer besides releasing some ions in the soil systems^12^. The reduction in sand and silt levels and simultaneous increase in clay contents as revealed in this study could be related with improved EC and WHC in the treated soil. This observation in changes of soil texture is corroborated with the findings of Baruah et al*.*^12^. Besides, the loss of total organic carbon (TOC) contents from the contaminated soil found in this study may be due to the utilization of organic carbon by microorganisms as well as the release of carbon in the form of CO_2_ during microbial respirations which become more accelerated in the presence of plants^20^ and thus there was higher loss of TOC in the soil where *Cyperus brevifolius* was introduced. Similarly, better improvement in pH, EC and WHC levels in T3 where the contaminated soils was treated with *Cyperus brevifolius* could be attributed to the synergistic actions of plant and associated microbial communities*.* It has been suggested that release of root exudates, hormones and other organic substances enhanced the microbial activities in the rhizosphere zone which in turn detoxify/reduce the intensities of crude oil pollutants and improves the soil physico-chemical conditions^6^. The decrease in total KJELDHAL nitrogen (TKN) contents may be attributed to the utilization of nitrogen by microorganisms to degrade organic pollutants present in soil. Additionally, greater loss of nitrogen along with carbon may be due to carbon mineralization and hydrocarbon immobilization resulting in excessive microbial activities using carbon materials as an energy source and its attendant demand for more nitrogen^1^. Different physiological and enzymatic processes in the soil systems help the plants to uptake nutrients. The increase in available phosphorus (AP) and total potassium (TK) may be related with poor uptake of these nutrients by plants. Moreover, the enhanced levels of AP and TK in the soil may also be related with the phosphate and potassium solubilizing microorganisms which are responsible for the increase in AP and TK although further planned study is needed to understand the reason.

The reduction in total oil and grease (TOG) contents indicates the potential of experimental plant for removal of hydrocarbons from the contaminated soil. This finding agrees the previous works of Bordoloi et al*.*^21^ who have reported about the efficiency of *Axonopus compressus* for removal of oil and grease from the crude oil-contaminated soil. Similarly, Basumatary et al*.*^6^ have reported about 32.6–50.01% reduction in TOG contents from contaminated soil employing *Cyperus rotundus* during 180 days of experimental trials. The reduction in oil and grease contents may be associated with the microbial activities, growth of the plants more particularly effective penetration of the root systems in the contaminated soil. It has been suggested that root exudates which control the quality and quantity of microbial population in soil also alter plant metabolism during crude oil associated stress condition thereby promoting the degradation of hydrocarbons/oil in vegetated treatments^6^. Several studies have showed significant changes in TOG profiles in oil polluted soils after treatment by herbs/grasses^22–24^. Nevertheless, reduction in TOG levels (17.36%) in the present study was found comparatively less as against the previous works which can be because of the time duration of the experiment (60 days), besides the high concentration levels of crude oil of the experimental soil. For example, Muratova et al*.*^25^ reported up to 52% reduction in oil and grease contents during three years of rye cultivation. Additionally, Razmjoo et al*.*^26^ found 40% reduction in TOG levels by employing Bermuda grass by the end of six months of experimental trials.

#### Soil enzyme activity and beneficial bacterial population

The altered values of dehydrogenase, urease, alkaline phosphatase, catalase, amylase, peroxidase and polyphenol oxidase by end of the experimental trial have proved the effect of petroleum products on the activities of these soil enzymes. High level of crude oil contamination in soil can suppress the expression of enzyme activities^27,28^. Soil enzymes are good indicator of soil health. These are considered as natural mediators and catalysts of several important soil processes such as process of decomposition of organic matter, soil humus formation, released of mineral nutrients to the soil and their supply to plants, fixation of molecular nitrogen and the flow of the chemical elements of the biochemical cycle^29^. The enhanced level of enzyme activities of the present study could be corroborated with the previous work of Yenn et al*.*^30^, where the authors have reported about the higher level of dehydrogenase, urease and phosphatase activities during phytoremediation of crude oil contaminated soil. The introduction of plants probably accelerates the microbial activities in the contaminated soil which in turn enhanced the enzyme activities. Moreover, the rise in enzyme activities after the introduction of plants may be also linked with the higher level of oxygen in the rhizosphere zone which results in a higher population of aerobic microbes and this is even very true in case of enhanced catalase activity in soil^31^. Besides, it has been suggested that fluctuation in amylase activities in soil is correlated with the changes in water holding capacity, organic carbon, total nitrogen and available phosphorous^32^. On the other hand, the decrease in polyphenol oxidase and peroxidase activities in T3 samples could be attributed to the specific microbial group that was not flourished well in the soil system although further planned study is required to explore the proper reason. Similarly, it was reported that soil organic carbon levels is associated with changes in cellulase activities^33^ and thus marginal decrease in soil cellulase activities in T3 could be linked with the reduction in organic carbon contents after treatment by *Cyperus brevifolius.*

The increase in total population of nitrogen fixing, phosphate and potassium solubilizing bacteria has showed the conformity with earlier works^5,34^ where it was reported that 6.11–11 folds increase in beneficial microbial population is possible in the contaminated soil after phytoremediation trials. Thus enhanced level of these beneficial bacterial populations in vegetated treatment i.e. T3 could be related with the release of plant’s exudates, mineralization/addition of nutrients to the soil system besides degradation of hydrocarbons to different intermediates which may again act as the nutrients for the microbes. As reported by Abedi-koupai et al.^35^ plants impart root exudates that are rich in carbon, energy, enzymes and even sometimes oxygen to support large number of microbial populations in soil. Further, increase in beneficial bacterial population in non-vegetated treatment i.e. T2 may be due to the evaporation of some volatile contaminants which may offer less toxic environment to the microbial community. Nevertheless, higher increase in total population of beneficial bacteria in T3 than T2 shows the conformity with the previous work of Bank et al*.*^36^ who have reported that presence of plants greatly influence microbial population in soil systems than unplanted one.

### Changes in plant characteristics

#### Plant productivity parameters

Crude oil pollution creates unsuitable conditions in soil and adversely affects the productivity of plants. Thus plant’s productivity is one of the important indicators to understand the impact of crude oil pollution on plant^13^. The lower shoot dry biomass of *Cyperus brevifolius* in T3 than T4 as found in this study could be related to the low availability of nutrients/minerals and acidic conditions in the contaminated soil. Besides, water deficiency conditions in crude oil contaminated soil inevitably reduced leaf area index (LAI) and chlorophyll contents of *Cyperus brevifolius* in T3 resulting difficulty in transpiration and photosynthesis which in turn results in retarded plant growth and biomass^37,38^. Nevertheless, the greater dry biomass of root of *Cyperus brevifolius* in T3 is likely to be associated with the adaptive response of the plant under stress environment. It has also been suggested that more extensive and deep root systems are related to effective functioning for hydrocarbon rhizodegradation^36^. As a whole, the findings of this study are in conformity with the reports of earlier workers^39^ who have enumerated that higher hydrocarbon levels in contaminated soil create a stress environment and affect the overall growth of the plants.

#### *Plant’s *in vitro* antioxidant activity and phytochemical contents*

The plants that could survive in the contaminated habitats show some adaptive response to overcome the stress. Crude oil pollutants create an oxidative stress condition in plants and to mitigate these, plants uses their antioxidant defense systems^14^. The lower IC50 values for antioxidant under stress conditions indicate the higher free radical-scavenging activities of the plants^40,41^. The higher free radical-scavenging or the greater level of antioxidant activity in the plant samples obtained from T3 could be related with plant’s defense mechanism in crude oil polluted soil. The increase in DPPH free radical scavenging activity is an index of inhibition in lipid peroxidation which indicates the oxidative stress conditions in plants. The reducing power, DPPH, H_2_O_2_ free radical and other antioxidant interact with reactive oxygen species (ROS) or high level of free radicals to constraint oxidative stress (OS) by inhibiting lipid peroxidation and improve strong resistance power by protecting potential cell injury^42^. Therefore high antioxidant capacity of *Cyperus brevifolius* indicates that it possesses a strong defense against the stress condition to prevent lipid peroxidation. Again higher value of phenol and flavonoid contents in the plant samples of T3 may be due to the higher antioxidant activities. There is well supported evidence that the phenolic compounds possess free radical scavenging properties and flavonoids are reported as superoxide (^**.**^O_2_^−^) and hydroxyl (^**.**^OH) radical scavengers^43,44^.

#### FT-IR analysis

FT-IR spectroscopic evaluation has confirmed the changes in intensities of bands and peaks in the plant samples obtained from T3 and T4. The presence as well as absence or changes in the intensities of bands and peaks for functional groups reflects the impact of crude oil pollution on the experimental plants. More intense bands representing the different functional groups such as phenols, flavonoids, aliphatic hydrocarbons, aromatics, carboxylic acids group and others in the plant samples obtained fromT3 has indicated the uptake/presence of petroleum hydrocarbons^1^. The increase and/or decrease as well as shifting in bands/peaks is possible when plants are exposed to hydrocarbons associated stress and uptake/metabolize the hydrocarbon components resulting from crude oil contaminations^1,45^. The presence of a more intense band of C–H stretching of methylene (–CH_2_–) in the sample of T3 clearly indicates the uptake of this compound from oil contaminated soil^46^. Again, presence of a clear and dominant band of C–O stretching of alkyl aryl ethers such as anisole (a benzene derivative) in T3 may be associated with uptake of this hydrocarbon compound by the experimental plant from crude oil contaminated soil. Further, the presence of carbonyl compounds such as aldehydes (C=O stretching) could be attributed to microbial oxidation process of used oil in the rhizosphere^46^. The presence of alcohols, aliphatic chloride, iodide, ethers and others in both the samples but with sharp and strong intensity spectra in T4 treatment justify that these compounds are building blocks of plants^47^.

## Conclusion

The herb species *Cyperus brevifolius* shows significant changes in antioxidant and phytochemical profiles to adapt in the aged crude oil contaminated soil. The altered values of antioxidant and phytochemical contents along with physico-chemical properties clearly confirm that the herb species *Cyperus brevifolius* have the potential to adapt with the stress condition induced due to crude oil contamination. The species is able to decrease the total oil and grease concentrations in the contaminated soil leading in improvement of soil biological properties that includes enhancement in various enzymatic activities such as dehydrogenase, urease, catalase, alkaline phosphatase and amylase along with increase in nitrogen fixing, phosphate and potassium solubilizing bacterial population. Again, FT-IR spectra confirm that *Cyperus brevifolius* has ability to uptake and metabolize some possible hydrocarbon components from polluted environment. Hence, the herb *Cyperus brevifolius* is a good candidate with better adaptive responses for adopting in practical approaches for remediation programmes of crude oil/hydrocarbon contaminated soil.

## Materials and methods

### Soil collection and experimental plant

The soil samples for the experiment were collected from Lakowa oil field (25°01´NL and 94°50´EL) of Assam, India. About 200 kg of heavily contaminated surface soil (depth 0–15 cm) from the agricultural field was collected for the experimental use. Immediate analysis of soil samples were done for total oil and grease/hydrocarbon contents to understand the intensity of oil contamination. The analysis revealed that average value of total oil and grease/hydrocarbons in the soil samples was 111.333 ± 5.271 g/kg (Table 1) which indicates that soil system is heavily polluted with crude oil as per the Dutch criterion (i.e. hydrocarbons concentration beyond 5800 mg/kg or 5.8 g/kg) of hydrocarbon pollution level^48^. Further, textural analysis of the collected soil samples revealed that it was sandy loam in nature (Table 1).The soil samples were shade dried, removed unwanted materials and finally sieved (mesh size 2 mm) before use in the experimental trials.

The herb species *Cyperus brevifolius* was selected as the experimental plant for the study. The *Cyperus brevifolius* was found to grow abundantly in crude oil contaminated sites of Assam^5,10^. The seeds of this plant were collected locally from Gauhati University campus, Guwahati, Assam India and grown in soil bed and seedlings of average size of 13.08 ± 0.07 cm height containing 7 ± 0.51 numbers of leaves was transferred into the experimental pots to carry out the study.

### Experimental setup

The experiment was carried out in plastic pots in net house under natural condition during the month of April–May in 2018. The temperature ranges within 28–33 °C during the entire duration of the experimental period. Finally processed soil measuring 250 g was taken in the experimental pots separately, added with distilled water and kept in shade for three days before the introduction of the plant seedlings. Two treatments were taken during the experiment—the first one was carried out in contaminated soils and the second one was carried in non-contaminated soil mainly to compare the different plant growth parameters in contaminated and normal soil conditions. A control set up of contaminated soil was also maintained without plants. The details of the experimental treatments are as follows-

T1- Crude oil contaminated soil (Initial); T2- Crude oil contaminated soil without *Cyperus brevifolius*; T3- Crude oil contaminated soil + *Cyperus brevifolius*; T4- Non-contaminated soil + *Cyperus brevifolius*; Ti- seedlings of *Cyperus brevifolius* grown in soil bed initially for the study*.*

The duration of the experiment was fixed for 60 days. Each pot was provided with a single plant and ten replicas were taken at the start of the experimental trials. Survivability of the transplanted seedlings was monitored and the seedlings which were unable to survive were removed immediately from the respective pots. Finally, the survivability percentages were calculated. Physico-chemical characteristics, total oil and grease (TOG) contents, enzyme activity and beneficial bacterial population in the soil were studied at the beginning and after harvest of the plants. Further, antioxidant profiles, phytochemical contents and productivity parameters along with functional groups status of the experimental plant were also investigated during the experimental trials.

### Analysis of soil samples

Physico-chemical characteristics including pH, conductivity, water holding capacity (WHC), soil texture, total organic carbon (TOC), total KJELDAHL nitrogen (TKN), available phosphorus (AP), total potassium (TK) of the soil samples were analyzed by standard methods^49,50^. For the study on physico-chemical characteristics, the air dried soil samples were taken. The pH and conductivity were measured in 1:5 (w/v) soil and water suspension with the help of digital pH (Biochem PM79) and conductivity meter (Systronics 304) respectively. The WHC were determined using Keen Raczkowski box technique following the method of Piper^51^. In brief, the soils were packed by tapping the boxes 15 times on a table after placing circular filter papers on the perforated bottom of the Keen Raczkowski boxes. The soil samples were leveled to the top of the boxes and kept overnight in water (up to 1 cm depth) in a tray. The boxes with more or less water saturated soil were then weighted, dried in a hot air oven at 95 °C until a constant weight was obtained. Finally, the WHC were estimated using the formula as described by Piper^50^. Soil texture was measured in sieve shaker following the method outlined by Trivedy and Goel^52^. Walkley and Black titration method as described by Jackson^53^ was employed to measure the TOC content in the samples. TKN in the samples was measured by micro KJELDAHL method^53^. The AP contents of soil samples were determined following the stannous chloride method^50^ by using the spectrophotometer (Shimadzu UV 1601). TK was analyzed by acid digestion method using flame photometer with a standard solution^50^. Total oil and grease (TOG) contents of the soil samples were determined by soxhlet extraction method using a modification of EPA method 3540B, taking dichloromethane (DCM) as the solvent and measured gravimetrically^6,54^.

Analysis of soil enzymes including dehydrogenase, urease, polyphenol oxidase, amylase, cellulase, peroxidase, catalase and alkaline phosphatase activities were done by following the standard methods. Dehydrogenase activity was analyzed by the method of Casida et al.^55^. Hoffman and Teicher^56^ method was used to determine the urease activity of the samples. The alkaline phosphatase activity was analyzed by the standard method of Tabatabai and Bremner^57^. Catalase activity was measured by employing the method of Johnson and Temple^58^. The methods of Cole^59^ and Pancholy and Rice^60^ were used to measure amylase and cellulase activities respectively. Polyphenol oxidase and peroxidase activities were determined by the standard protocols as described by Bach et al*.*^61^ and German et al*.*^62^^.^

The beneficial bacterial population that includes total nitrogen fixers, phosphate and potassium solubilizers were enumerated in Jensen’s medium, Pycovskaya’s and Aleksandrow agar respectively following the standard protocols as described by Kaundal et al.^63^ and Etesami et al.^64^. The colonies appeared after incubating the petriplates for 2–3 days at 35 ± 2 °C in a bacteriological incubator were selected for enumeration. The photographs of the petriplates were taken by Nikon D780 camera and the number of colonies were counted using OpenCFU software.

### Analysis of plant samples

Analysis of plant parameters includes the antioxidant assay such as 1,-1-diphenyl-2-picrylhydrazyl (DPPH) free radical scavenging, hydrogen peroxide (H_2_O_2_) radical scavenging, reducing power assay; besides total phenol and flavonoid contents, productivity parameters and FT-IR spectroscopic evaluation for functional group studies. For the antioxidant and phytochemical assay, the harvested plant samples were shade dried and then grinded into fine powder. The powdered samples were dissolved in methanol in the ratio of 1:10 (w/v) and kept in a shaking incubator for 24 h at 180 rpm maintaining the temperature at 30 °C. The solutions obtained were filtered with Whatmann No.1 filter paper and the filtrate/extract were used for determination of DPPH and H_2_O_2_ free radical scavenging activity as well as reducing power assay along with total phenol and flavonoid contents following the standard protocols. DPPH radical scavenging effects of the plant extract were analyzed by the method of Brand-Williams et al*.*^65^. The H_2_O_2_ radical scavenging activity was determined by employing the method as outlined by Ruch et al*.*^66^. Reducing power was measured by the method of Oyaizu^67^. Total phenol and flavonoid contents of the extract were estimated using the methods of Hagerman^68^ and Jay et al*.*^69^ respectively. The plant productivity parameters were analyzed in terms of Leaf area index (LAI), estimation of chlorophylls and dry biomass as per standard method outlined by Baruah et al*.*^13^.

The Fourier transform infrared (FT-IR) spectra of the experimental plant samples were monitored by FT-IR spectrophotometer (Nicolet 6700) as per the protocol described by Haghollahi et al*.*^70^. For the FT-IR analysis also, the harvested plant samples were dried in shade for three days then grinded into fine powder. The powdered plant samples (1 mg of each) were mixed with 100 mg KBr (Potassium bromide) in the ratio of 1:100, homogenized in an agate mortar, pressed and made into pellets, and finally spectra were taken in mid-infrared area within the range of 4000–400 cm^−1^.

### Statistical analysis

SPSS software (2018 version) was used for statistical analysis. The significant differences in the values of the soil and plant samples for different parameters were determined by paired t-test, one way ANOVA analysis and LSD test. For each analysis three replicas (randomly chosen for each treatment) were assayed.

## Supplementary Information

Supplementary Figures.
