# Supplementary material for: Enzymatic defense of Cyperus brevifolius in hydrocarbons stress environment and changes in soil properties
Source: Sci Rep. 2021 Jan 12;11:718. doi: 10.1038/s41598-020-80854-5 (PMC7804112; doi:10.1038/s41598-020-80854-5)
Supplement: Supplementary file 1 — Supplementary Figures. [file 41598_2020_80854_MOESM1_ESM.pdf]

Enzymatic defense of *Cyperus brevifolius* in hydrocarbons stress environment and changes in soil properties

Paramita Chakravarty and Hemen Deka*

Environmental Botany and Biotechnology Laboratory, Department of Botany, Gauhati University,

Guwahati-14, Assam, India

* Corresponding author

E-mail address: hemendeka@gauhati.ac.in/ dekahemen8@gmail.com (H. Deka).

Tel: +918638689332 (M); fax: +913612700311

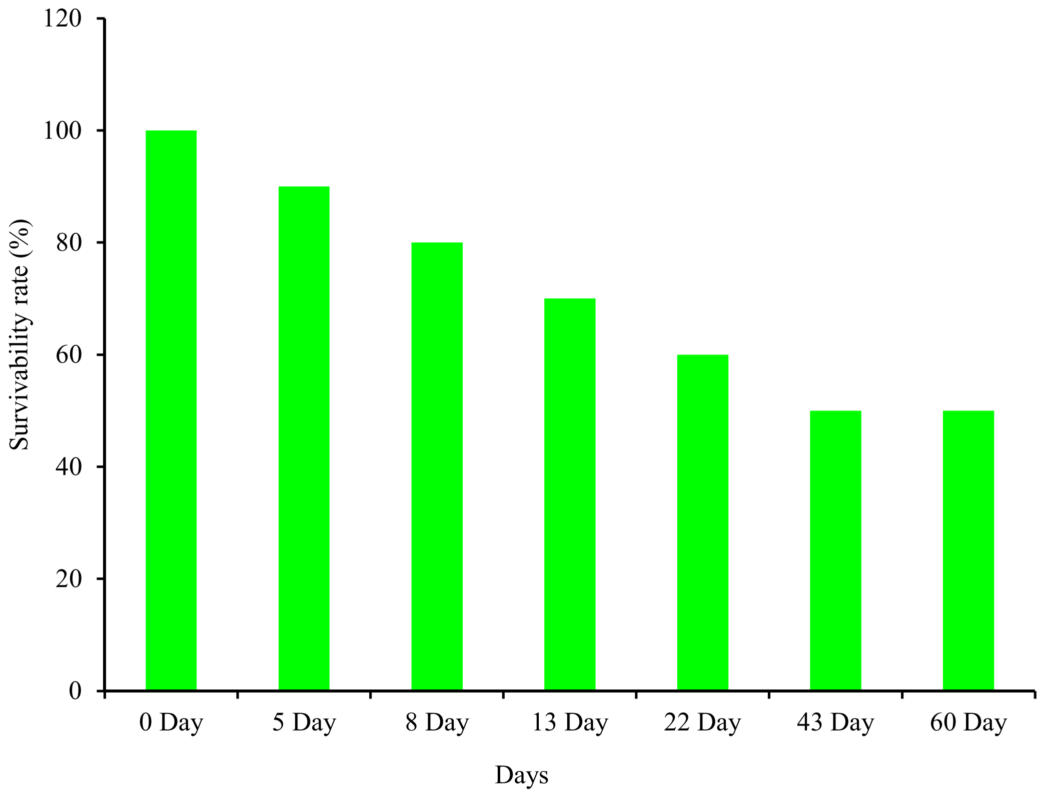

Figure 1: Showing survivability rate of *Cyperus brevifolius* on crude oil-polluted soil

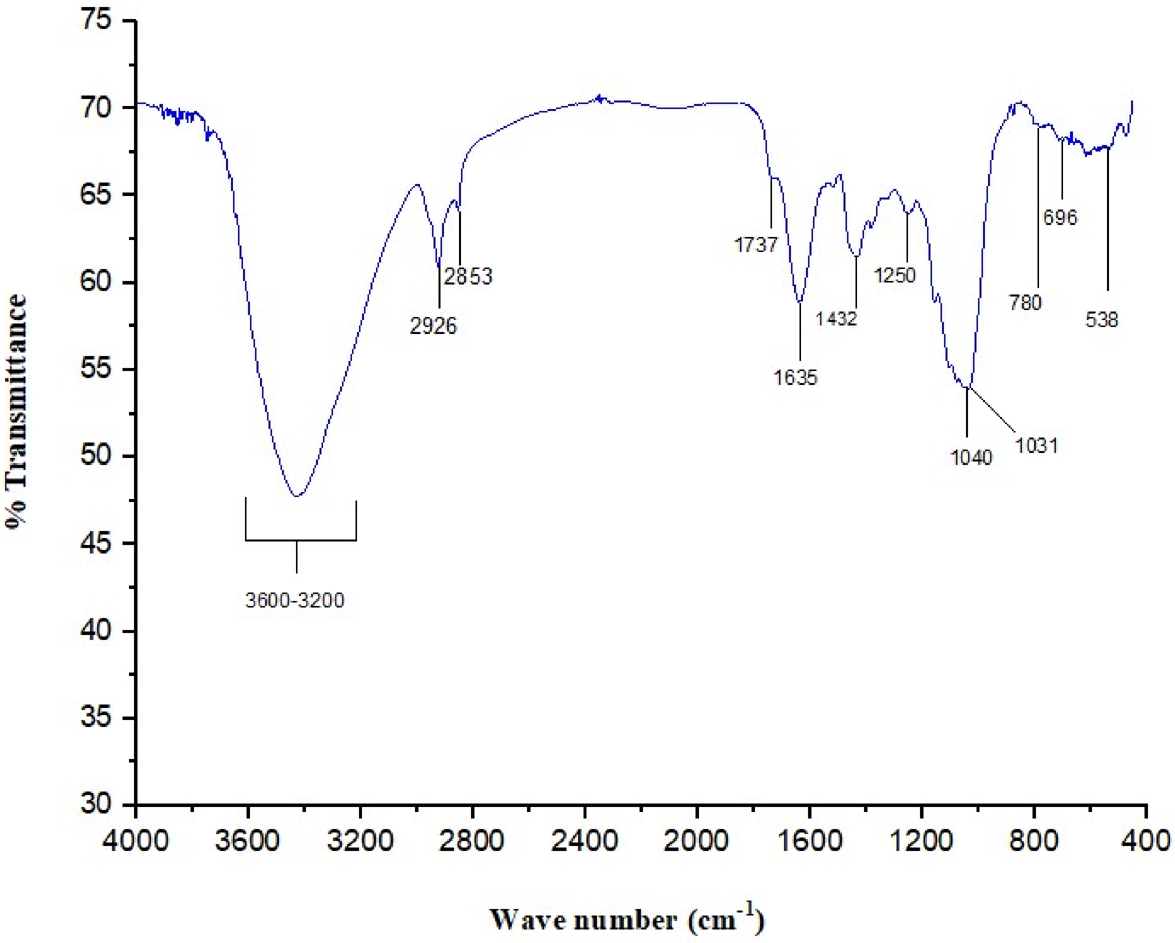

Figure 2: FT-IR spectra of plant sample from T3 treatment

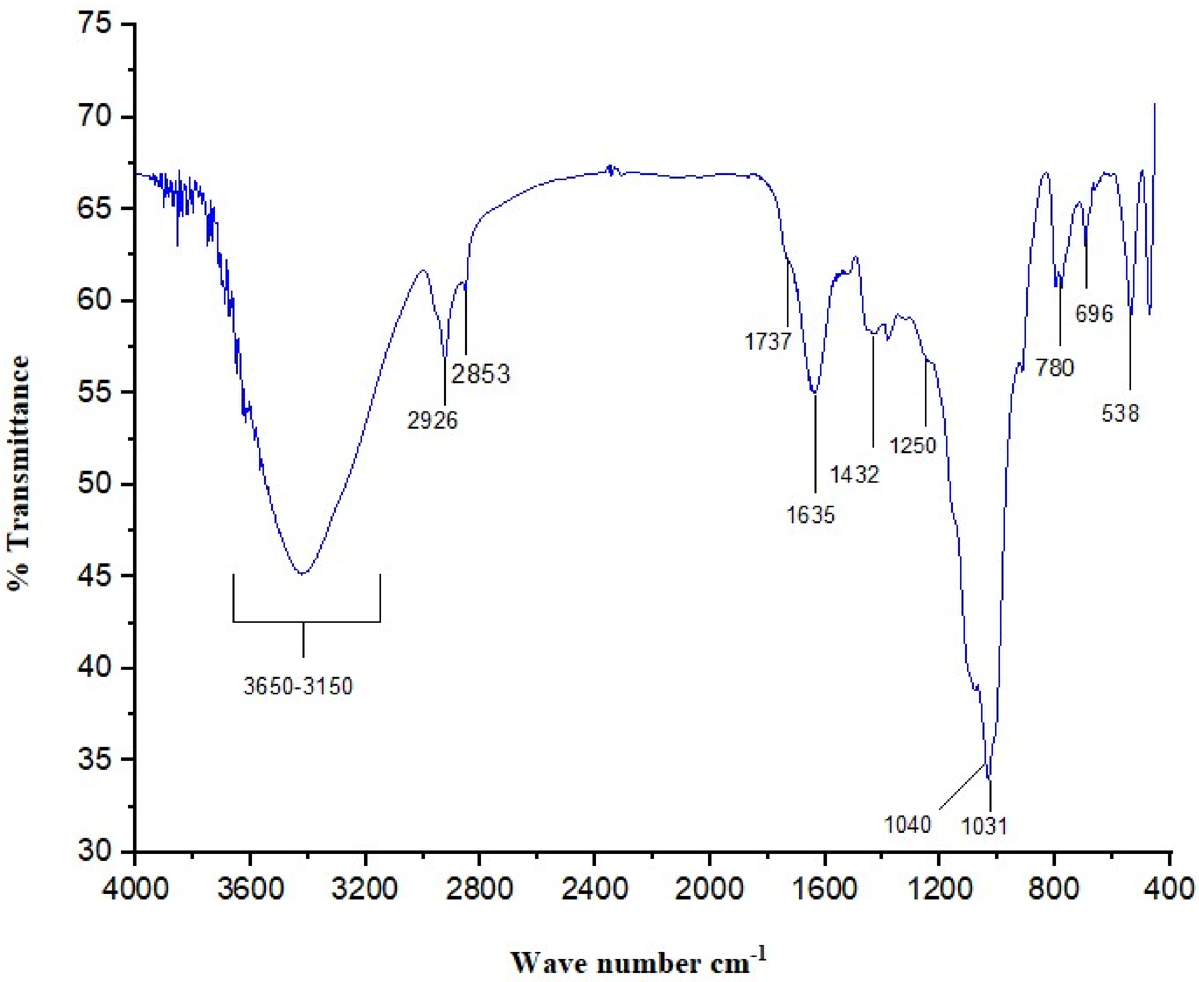

Figure 3: FT-IR spectra of plant sample from T4 treatment
